# Novel Diels–Alder Type Adducts from *Morus alba* Root Bark Targeting Human Monoamine Oxidase and Dopaminergic Receptors for the Management of Neurodegenerative Diseases

**DOI:** 10.3390/ijms20246232

**Published:** 2019-12-10

**Authors:** Pradeep Paudel, Se Eun Park, Su Hui Seong, Hyun Ah Jung, Jae Sue Choi

**Affiliations:** 1 Department of Food and Life Science, Pukyong National University, Busan 48513, Korea; phr.paudel@gmail.com (P.P.); gogo1685@naver.com (S.E.P.); seongsuhui@naver.com (S.H.S.); 2 Department of Food Science and Human Nutrition, Jeonbuk National University, Jeonju 54896, Korea

**Keywords:** dopamine, GPCRs, human monoamine oxidase, *Morus alba* L., Parkinson’s disease

## Abstract

In this study, we delineate the human monoamine oxidase (*h*MAO) inhibitory potential of natural Diels–Alder type adducts, mulberrofuran G (**1**), kuwanon G (**2**), and albanol B (**3**), from *Morus alba* root bark to characterize their role in Parkinson’s disease (PD) and depression, focusing on their ability to modulate dopaminergic receptors (D_1_R, D_2L_R, D_3_R, and D_4_R). In *h*MAO-A inhibition, **1**–**3** showed mild effects (50% inhibitory concentration (IC_50_): 54‒114 μM). However, **1** displayed moderate inhibition of the *h*MAO-B isozyme (IC_50_: 18.14 ± 1.06 μM) followed by mild inhibition by **2** (IC_50_: 57.71 ± 2.12 μM) and **3** (IC_50_: 90.59 ± 1.72 μM). Our kinetic study characterized the inhibition mode, and the in silico docking predicted that the moderate inhibitor **1** would have the lowest binding energy. Similarly, cell-based G protein-coupled receptors (GPCR) functional assays in vector-transfected cells expressing dopamine (DA) receptors characterized **1**–**3** as D_1_R/D_2L_R antagonists and D_3_R/D_4_R agonists. The half-maximum effective concentration (EC_50_) of **1**–**3** on DA D_3_R/D_4_R was 15.13/17.19, 20.18/21.05, and 12.63/‒ µM, respectively. Similarly, **1**–**3** inhibited 50% of the DA response on D_1_R/D_2L_R by 6.13/2.41, 16.48/31.22, and 7.16/18.42 µM, respectively. A computational study revealed low binding energy for the test ligands. Interactions with residues Asp110, Val111, Tyr365, and Phe345 at the D_3_R receptor and Asp115 and His414 at the D_4_R receptor explain the high agonist effect. Likewise, Asp187 at D_1_R and Asp114 at D_2L_R play a crucial role in the antagonist effects of the ligand binding. Our overall results depict **1**–**3** from *M. alba* root bark as good inhibitors of *h*MAO and potent modulators of DA function as D_1_R/D_2L_R antagonists and D_3_R/D_4_R agonists. These active constituents in *M. alba* deserve in-depth study for their potential to manage neurodegenerative disorders (NDs), particularly PD and psychosis.

## 1. Introduction

Monoamine oxidase (MAO) is a flavoenzyme in the outer mitochondrial membrane of neuronal and non-neuronal cells that has a vital role in the etiology of age-regulated neurodegenerative disorders (NDs). MAO catalyzes the oxidative deamination of monoamine neurotransmitters, dietary amines, and xenobiotics and regulates their levels and functions in the brain. During oxidative deamination, MAO liberates hydrogen peroxide, the reactive oxygen species (ROS) most potent in causing oxidative stress and mitochondrial dysfunction [1]. Though the etiology of NDs remains unclear, apoptosis, oxidative stress, mitochondrial dysfunction, inflammation, an impaired ubiquitin-proteasome system, and excitotoxicity are common disease-modifying factors [2]. Two isoforms (MAO-A and MAO-B) with specific functions have been identified in different brain regions and cell types [3].

**Figure 1:**
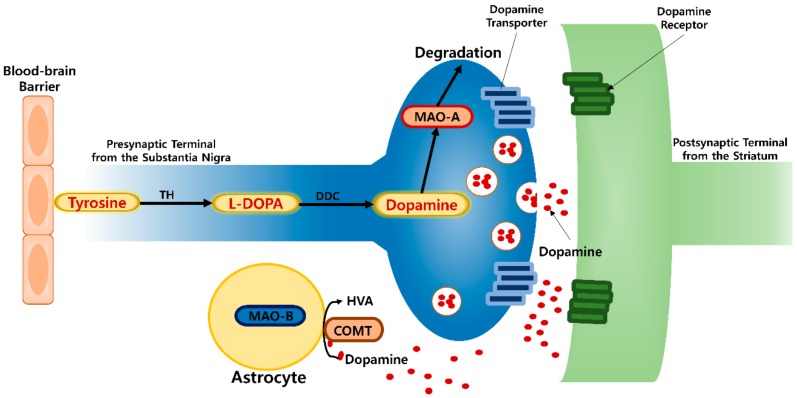
Activity of monoamine oxidase (MAO) enzyme in neuronal cells.

MAO-A displays a higher affinity for serotonin (5HT) and norepinephrine, whereas MAO-B prefers phenylethylamine. Dopamine (DA) and tyramine are common substrates for both isozymes [4]. MAO-A is associated with the onset of psychiatric disorders (Figure 1), including depression, and antisocial aggressive impulsive behaviors through its ability to decrease neurotransmitter levels (DA and serotonin) [5,6]. During a normal physiological state, DA levels in substantia nigra pars compacta (SNpc) are regulated as an equilibrium between synthesis, synaptic vesicle loading, uptake, and catabolism. MAO enzyme mediates oxidative deamination of DA to DOPAL along with H_2_O_2_ generation, leading DA deficit and oxidative stress state. And MAO-A inhibition prevents the deamination of neurotransmitters, reduces oxidative stress, and increases the availability of neurotransmitters within noradrenergic and serotonergic neurons of the CNS to regulate neuron signaling via their respective receptors [4,7]. Similarly, MAO-B metabolizes DA to DOPAC and catechol-O-methyltransferase (COMT) degrades it to homovanillic acid (HVA) in astrocyte [8,9]. Therefore, MAO inhibitors function as neuroprotective agents against age-related NDs.

The concept of precision medicine relies on protein targeting, and G protein-coupled receptors (GPCRs) are the largest family of target receptors and membrane proteins. At present, 34% of FDA-approved drugs target GPCRs [10]. GPCRs are widely expressed and activated by a broad range of ligands, including neurotransmitters, hormones, and ions, as well as sensory signals [11]. Neurotransmitters bind to their specific receptors at the postsynaptic cleft and trigger or inhibit neuronal functions and signals by regulating the activity of ion channels. In NDs, especially Parkinson’s disease (PD), the selective loss of dopaminergic neurons in the SNpc produces DA deficiency, which triggers cell-specific alterations in intrinsic excitability and synaptic plasticity [12]. Therefore, regulating DA levels or DA receptor signaling is a standard approach to PD treatment. Numerous neurotransmitters and their analogs have therapeutic properties, serve as medicaments for various diseases, and have been the subject of extensive pharmacological studies [13]. In this study, we discuss the critical physicochemical interactions between our test ligands and different residue side chains and the adjacent amino acids.

**Figure 2:**
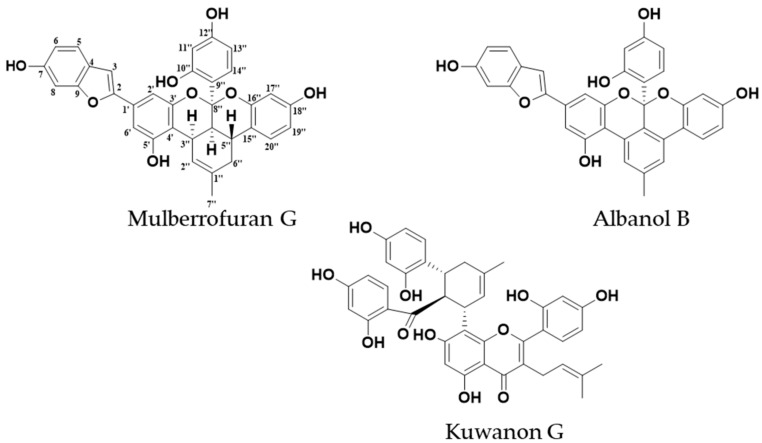
Structures of compounds isolated from *Morus alba.*

*Morus alba* Linn, commonly known as mulberry, is a perennial woody plant of the family Moraceae that is widely cultivated in tropical, subtropical, and temperate zones in Asia, Europe, and North and South America. The leaves of this plant are used as feed for animals and sericulture, the fruit is used as food, and the wood as timber. Furthermore, in traditional Chinese medicine, the leaves, twigs, fruit, and root bark are used as antioxidant, anti-inflammatory, anti-hypertensive, hypoglycemic, immunomodulatory, hypolipidemic, antibacterial, and anti-tumor agents [14]. The plant thus has unique medicinal and ethnic values. It is rich in flavonoids, alkaloids, steroids, and coumarins. Diels–Alder-type adducts are prototypical metabolites in the root bark [15]. In a previous study, mulberry fruit extract protected dopaminergic neurons in in vitro and in vivo PD models by regulating ROS generation through its antioxidant and anti-apoptotic effects [16]. A crude water extract of *M. alba* leaf ameliorated alterations in the retinal neurotransmitters adrenaline, DA, gamma-aminobutyric acid, histamine, noradrenaline, and serotonin in the pups of diabetic and hypercholesterolemic mother rats [17] and ameliorated kidney damage in diabetic rats by suppressing inflammation and fibrosis via peroxisome proliferator-activated receptor γ (PPARγ) modulation [18]. Similarly, a leaf-ethanol extract possessed anxiolytic and muscle-relaxant activities, probably via a γ-aminobutyric acid A-benzodiazepine (GABA_A_-BZD) mechanism [19]. No previous reports have considered the root bark of *M. alba.* In our recent work, we reported the antidiabetic [20,21], anti-Alzheimer’s disease activity [22,23], and antioxidant and anti-browning property [24] of Diels–Alder-type adducts and arylbenzofurans from *M. alba* root bark. More recently, kuwanon G and albanin G from the root bark were hypothesized as the components responsible for the appetite suppression activity of root-bark extract via cannabinoid (CB1) receptor antagonism [25]. In the present study, we characterize the multi-target effects of Diels–Alder-type adducts, mulberrofuran G (**1**), kuwanon G (**2**), and albanol B (**3**) (Figure 2), via human monoamine oxidase (*h*MAO) inhibition and the modulation of dopaminergic receptors (D_1_R, D_2_R, D_3_R, and D_4_R), and we use a molecular simulation to explore the action mechanism of the ligand–receptor interaction.

## 2. Results

### 2.1. In Vitro hMAO Inhibition and Enzyme Kinetics

The in vitro *h*MAO inhibition potentials of **1**–**3** and the reference compound selegiline was evaluated via a chemiluminescent assay in a white, opaque, 96-well plate using the MAO-Glo kit (Promega, Madison, WI). At first, **1**–**3** were screened for *h*MAO activity at 100 µg/mL and the % inhibition was 93.87%, 99.05%, and 74.85%, respectively. Then the compounds were retested at different micromolar concentrations in triplicates and the 50% inhibitory concentration (IC_50_) values obtained from the log-dose inhibition curve are tabulated in Table 1.

As shown there, **1**–**3** displayed mild inhibition of *h*MAO-A activity. Among the test compounds, **1** showed the best inhibition, with an IC_50_ value of 54.79 ± 0.03 µM, followed by **2** (IC_50_: 70.16 ± 2.60 µM) and **3** (IC_50_: 114.31 ± 2.30 µM). The inhibition potentials of **1**–**3** were better against *h*MAO-B, though the pattern of inhibition was similar**: 1** showed moderate inhibition effect, with an IC_50_ value of 18.14 ± 1.06 µM, and compounds **2** and **3** mildly inhibited *h*MAO-B, with IC_50_ values of 57.71 ± 2.12 and 90.59 ± 1.72 µM, respectively. The reference inhibitor selegiline inhibited the activity of isozymes -A and -B at IC_50_ values of 12.51 ± 1.11 and 0.30 ± 0.01 µM, respectively. However, compared to the reference reversible *h*MAO-A inhibitor (harmine, IC_50_: 0.006 µM) [26] and reversible *h*MAO-B inhibitor (safinamide, IC_50_: 0.00512 µM) [27], the potency of **1**‒**3** is significantly weaker.

**Figure 3:**
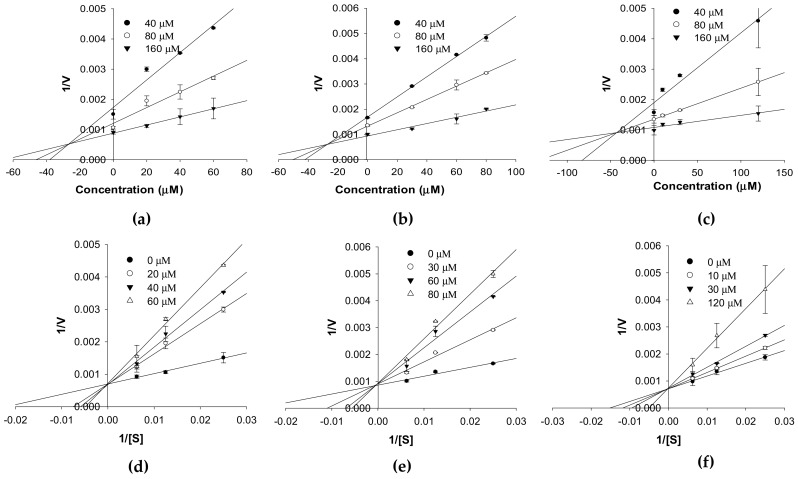
Dixon plot (**a‒c**) and Lineweaver–Burk plot (**d‒f**) of *h*MAO-A inhibition by compounds **1**–**3**, respectively.

**Figure 4:**
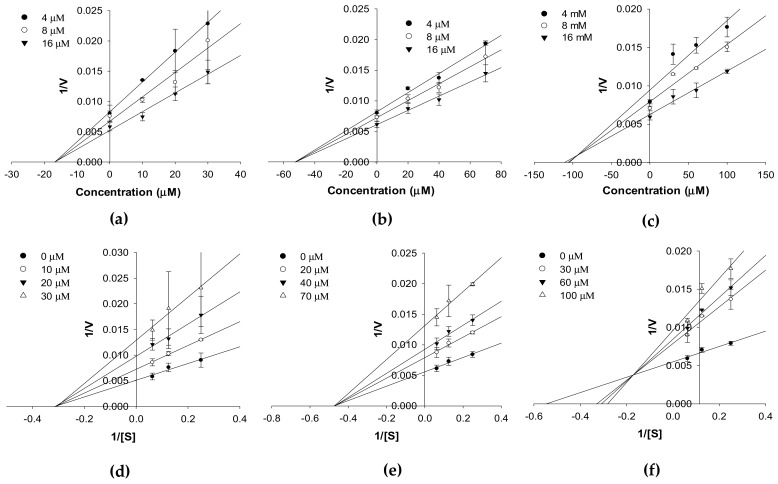
Dixon plot (**a‒c**) and Lineweaver–Burk plot (**d‒f**) of *h*MAO-B inhibition by compounds **1**–**3**, respectively.

The enzyme inhibition patterns of compounds at different substrate concentrations in the kinetic study are tabulated in Table 1 and represented in Figure 3 and Figure 4. Compounds **1**–**3** competitively inhibited *h*MAO-A isozyme activity with *K*_i_ values of 26.96 ± 3.98, 28.29 ± 2.02, and 46.93 ± 4.12 µM, respectively (Table 1 and Figure 3a–c). The Lineweaver–Burk plots (1/*V* vs. 1/[S]) for *h*MAO-A isozyme activity (Figure 3d–f) reveal an increase in *K*_m_ with an increase in the concentrations of **1**–**3**, whereas 1/*V*_max_ remained constant. Meanwhile, **1** and **2** were noncompetitive inhibitors (*V*_max_ value decreased in a concentration-dependent manner without changing the *K*_m_ value), and **3** was a mixed type inhibitor (increase in inhibitor concentration increased the *K*_m_ value but decreased the *V*_max_ value) of the *h*MAO-B isozyme (Figure 4c,f). From a Secondary plot (plot not shown here), the binding constants of **3** with a free enzyme (*K*_ic_) and with enzyme-substrate complex (*K*_iu_) identified were 55.19 ± 7.97 and 186.2 ± 10.26 µM, respectively. Likewise, the *K*_i_ value of **1** and **2** for *h*MAO-B inhibition was 17.01 ± 3.31 and 52.09 ± 5.56 µM, respectively.

### 2.2. In Silico Docking Simulation of hMAO

**Figure 5:**
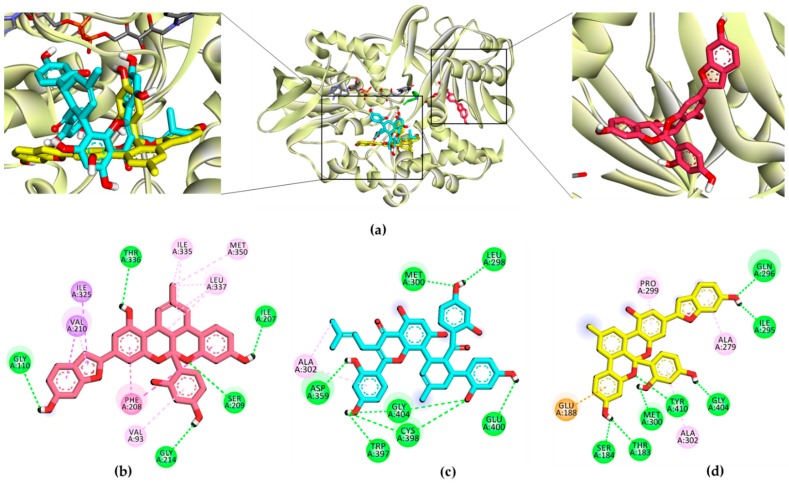
(**a**) *h*MAO-A inhibition mode of **1**–**3** and selegiline. (**b–d**) 2D ligand interaction diagram of *h*MAO-A inhibition by **1**–**3**, respectively.

**Figure 6:**
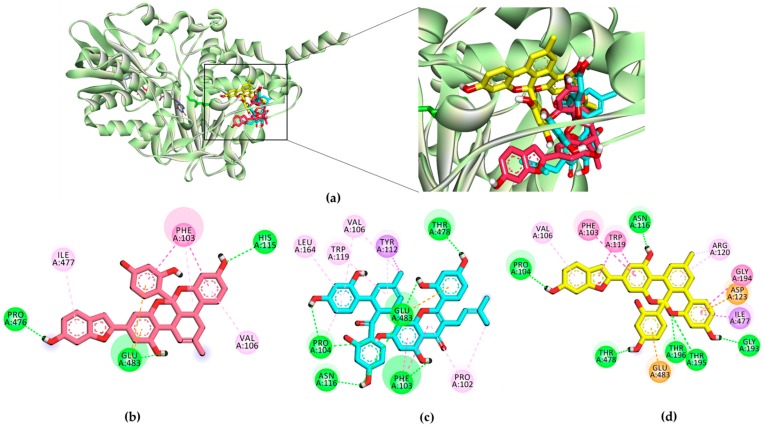
(**a**) *h*MAO-B inhibition mode of **1**–**3** and selegiline. (**b–d**) 2D ligand interaction diagram of *h*MAO-B inhibition by **1**–**3**, respectively.

**Figure 7:**
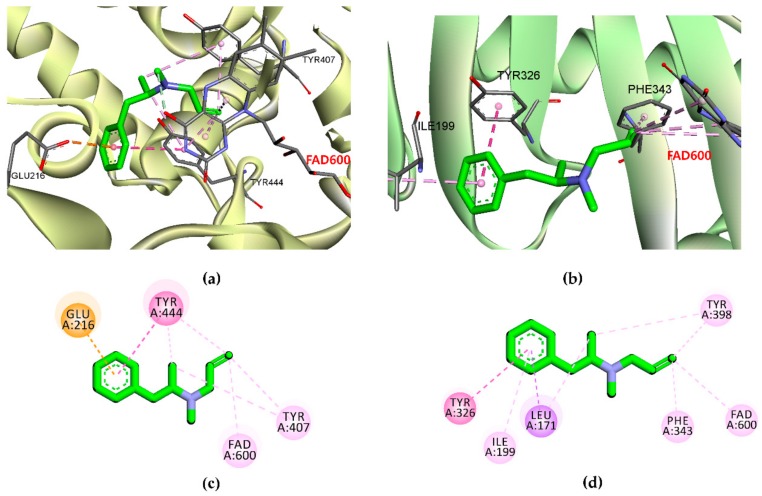
(**a**) *h*MAO-A and (**b**) *h*MAO-B inhibition mode of selegiline with flavin adenine dinucleotide (FAD). (**c**,**d**) 2D ligand interaction diagram of *h*MAO-A and *h*MAO-B inhibition by selegiline.

Computational modeling was performed to obtain insights into the binding affinity between ligands and the enzyme using AutoDock 4.2. To validate the docking result, the reference inhibitor selegiline as well as reversible inhibitor harmine (for *h*MAO-A) and safinamide (for *h*MAO-B) were docked into the active site cavity of the *h*MAO enzyme, and the ligands were re-docked. The results of the simulation study are tabulated in Table 2 and Table 3 and represented in Figure 5, Figure 6 and Figure 7.

As shown in Table 2, the test ligand (**1**–**3**)–*h*MAO-A complexes showed lower binding energies (−6.74 to −9.54 kcal/mol) than the reference ligand selegiline (−6.54 kcal/mol) and harmine (−6.46 kcal/mol). Ligand **1** posed in the active site by interacting with Gly110, Thr336, Ile207, Gly214, and Ser209 via a hydrogen bond (Figure 5). Meanwhile, ligands **2** and **3** shared Met300 and Gly404 as common H-bond interacting residues. Reversible inhibitor harmine interacted with flavin adenine dinucleotide (FAD)600, Ile335, and Tyr444 residues at the active site cavity, which were not observed for test ligand-binding. In the case of *h*MAO-B, ligands **1**–**3** showed high affinity with binding energies (−11.09, −12.65, and −10.05 kcal/mol, respectively) by forming three and five H-bond interactions, respectively (Figure 6). With the lowest binding energy, ligand **1** stably positioned in the *h*MAO-B active site by interacting with His115, Pro476, and Glu483 via H-bonds. Moreover, **1** interacted with peripheral residues, including Phe103, Val106, and Ile477. Interacting residues Val106 and Phe103 were shared by all three ligands as a noncompetitive inhibitor. Ligand **2** shared the most abundant H-bond interaction residues: Pro103, Asn116, Glu483, Phe103, and Thr478. Ligand **3** also showed high affinity via H-bond interactions with Thr195, Pro104, Asn116, Thr478, and Gly193. Selegiline interacted with Ile198 and safinamide with Ile199, Cys172, Tyr326, and Thr201 via H-bonds in the active site of *h*MAO-B (Figure 7).

### 2.3. Cell-Based Functional GPCR Assays

To characterize the possible role of compounds **1**–**3** in neuronal diseases, we first screened their functional activity at 100 µM on DA (D_1_, D_2_, D_3_, D_4_) receptors by measuring their effects on secondary messengers (cAMP modulation or Ca^2+^ ion mobilization) in transfected cell lines expressing human cloned receptors of interest. The data in Table 4 represent the agonist/antagonist effects of **1**–**3** at 100 µM on the various receptors.

**Figure 8:**
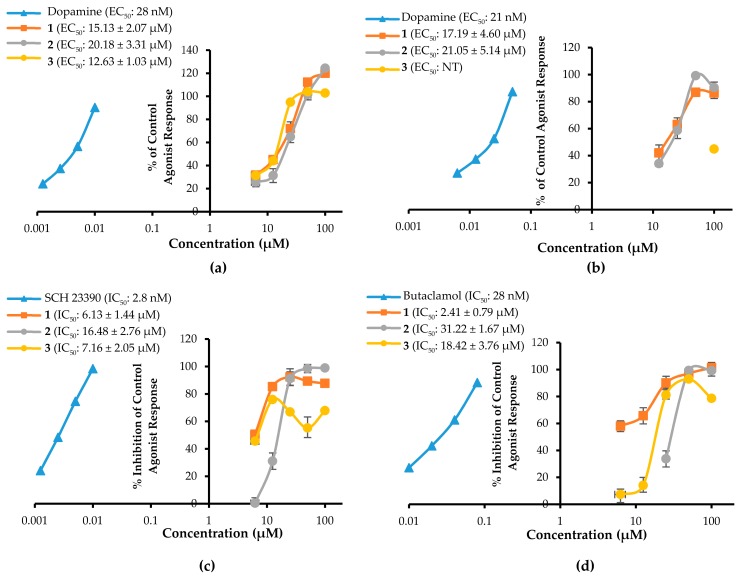
Concentration-dependent % of control agonist response on human dopamine D_3_ receptor (*h*D_3_R) (**a**) and human dopamine D_4_ receptor (*h*D_4_R) (**b**), and % inhibition of control agonist response on human dopamine D_1_ receptor (*h*D_1_R) (**c**) and *h*D_2L_R (**d**) of test compounds **1**–**3**.

As shown in the table, **1**‒**3** exhibited a full antagonist effect on the D_1_/D_2_ receptors and a full agonist effect on the D_3_/D_4_ receptors. The agonist effects of **1**–**3** at 100 µM on D_3_R/D_4_R were 119.9/86.30, 124.3/90.45, and 102.8/46.10%, respectively. Similarly, at 100 µM, **1**‒**3** inhibited the DA response on D_1_R/D_2L_R by 87.65/101.30, 98.85/99.15, and 67.80/78.55%, respectively. Figure 8 shows the concentration-dependent functional effect of **1**–**3** on the DA receptor subtypes with corresponding EC_50_/IC_50_ values.

As shown there, all compounds showed promising antagonist effects on D_1_R/D_2L_R, with IC_50_ values in the range of 2.41‒31.22 µM (Figure 8c,d). The rank order for the antagonist effect was D_2L_R > D_1_R for **1** and D_1_R > D_2L_R for **2** and **3**. Compared to **1** and **2**, the dose–response curve for **3** (Figure 8c) looks unusual due to relatively higher standard deviation in response at 50 µM concentration. Similarly, **1**‒**3** showed an agonist effect on D_3_R/D_4_R, with EC_50_ values in the range of 12.63‒21.05 µM (Figure 8a,b), and the rank order for the agonist effect was D_3_R = D_4_R for **1** and **2** and D_3_R > D_4_R for **3**. These results indicate that compounds **1**–**3** mediate the DA function by acting as D_1_R/D_2L_R antagonists and D_3_R/D_4_R agonists.

### 2.4. In Silico Docking Simulation of Dopamine Receptors

**Figure 9:**
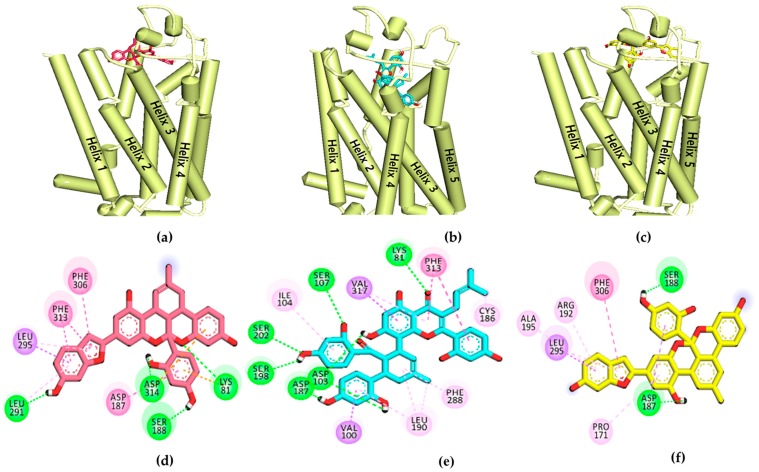
(**a–c**) Molecular docking simulation of **1**–**3** with human dopamine D_1_ receptor (*h*D_1_R). (**d–f**) 2D diagram of the ligand binding sites.

**Figure 10:**
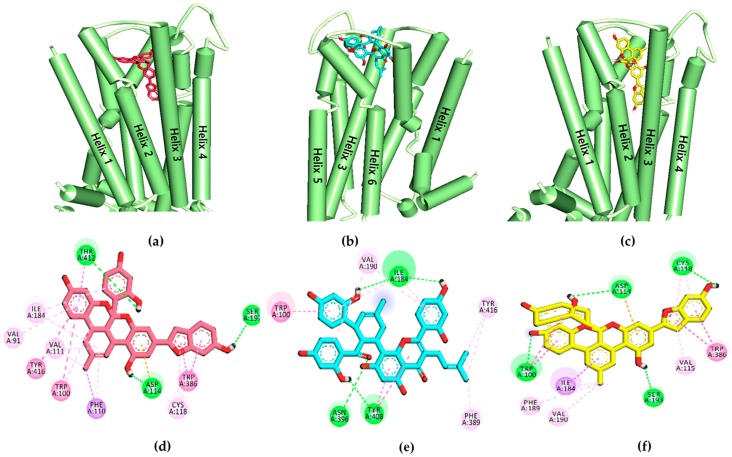
(**a–c**) Molecular docking simulation of **1**–**3** with human dopamine D_2L_ receptor (*h*D_2L_R). (**d–f**) 2D diagram of the ligand-binding sites.

**Figure 11:**
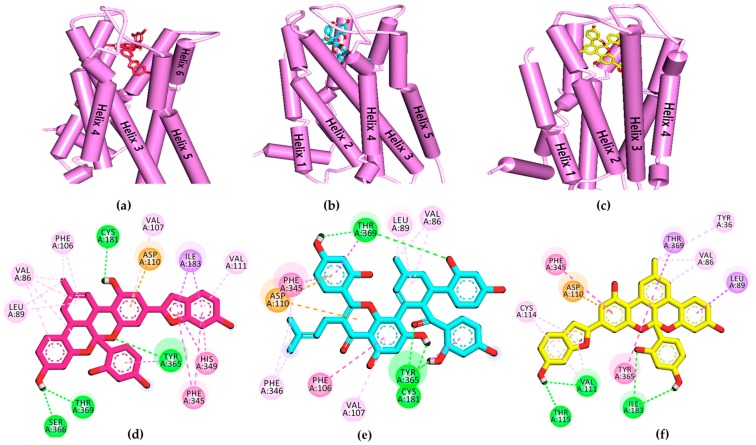
(**a–c**) Molecular docking simulation of **1**–**3** with human dopamine D_3_ receptor (*h*D_3_R). (**d–f**) 2D diagram of the ligand-binding sites.

**Figure 12:**
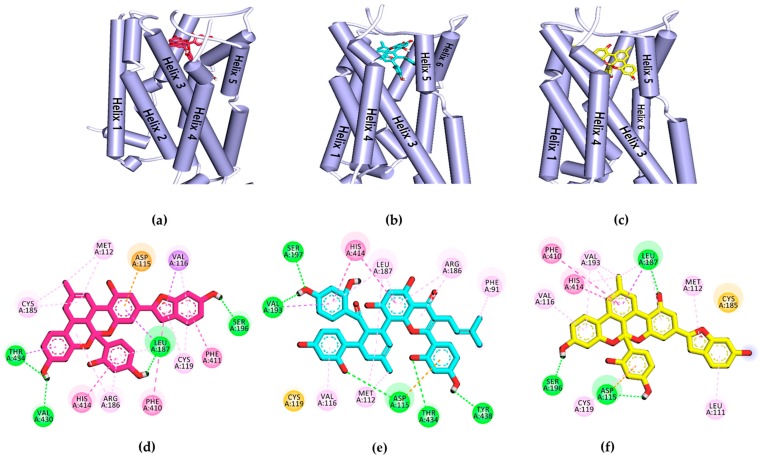
(**a–c**) Molecular docking simulation of **1**–**3** with human dopamine D_4_ receptor (*h*D_4_R). (**d–f**) 2D diagram of the ligand-binding sites.

To validate the results of the functional assays and investigate and identify the ligand–receptor interactions for novel lead discovery, we carried out a computational docking simulation using AutoDock 4.2 (Figure 9, Figure 10, Figure 11 and Figure 12). Since the effect of **1**‒**3** on DA receptors was promising, a simulation study was carried out on DA receptors. The binding affinities of reference ligands for each receptor were also evaluated to better understand and validate the docking results. The homology model of DA receptor subtype D_1_R was based on the structure of the β2-adrenergic receptor because it has a higher similarity to the DA D1 receptor in the binding site region and sequence identity [28]. Subtypes D_2L_R, D_3_R, and D_4_R were obtained from the protein data bank (PDB) IDs for 6CM4, 3PBL, and 5WIV, respectively. The results of the docking simulation are tabulated in Table 5, Table 6, Table 7 and Table 8 and represented in Figure 9, Figure 10, Figure 11 and Figure 12. The dotted colored lines in Figure 9, Figure 10, Figure 11 and Figure 12 represent specific interactions (green line: H-bond; purple line: π-sigma; pale pink: π-alkyl, alkyl; pink: π-π-T-shaped, π-π stacked; orange: π-anion).

As shown in Table 5, the **1**‒D_1_R complex exhibited four strong H-bond interactions with Lys81, Leu291, Ser188, and Asp314 with low binding energy (−9.22 kcal/mol). The ligand **2**‒D_1_R complex (−7.1 kcal/mol) interacted with Ser202 and Asp103 in H-bonds, similar to the reference antagonist SCH-23390 and agonist DA. Ligand **3**, with a binding energy of −9.2 kcal/mol, shared Ser188 and Asp187 with D_1_R via H-bonds. 

Furthermore, Asp187, Leu295, Phe306, Pro171, Ala192, and Ala195 were revealed as hydrophobic residues in the ligand **3**‒D_1_R complex (Figure 9). Figure 10 provides a close-up view of ligands **1**–**3** binding at the active site of D_2L_R. As shown in Table 6, ligands **1**–**3** bound strongly to the active site of D_2L_R with low binding energies (−8.11 to −10.45 kcal/mol). Risperidone and butaclamol are D_2L_R agonist and antagonist and they bound to the active site of the receptor with binding energies −12.7 and −6.9 kcal/mol, respectively, by forming salt-bridge with Asp114.

Though ligands **1**–**3** did not form a salt-bridge with Asp114, they showed H-bond and π-anion interactions with the residue. Furthermore, the number of H-bond interactions was higher for test ligands compared to resperidone and butaclamol.

Specifically, Asp114 was an H-bond and electrostatic residue for both ligands **1** and **3**, indicating high affinity with the receptor, whereas Ile184 was a crucially active residue in the second extracellular loop of D_2L_R, forming a π-alkyl interaction with **2**. Phe189 and Val190 are necessary key residues in antagonist-ligand binding, and they were well observed in the **2**‒D_2L_R and **3**‒D_2L_R complexes.

Among the test ligands, **3** showed the highest affinity (−10.41 kcal/mol) for ligand‒D_3_R interactions (Table 7). Ligand **1** had a slightly higher binding energy than **3** but was comparable to that of reference agonist DA (−6.9 kcal/mol). 

The key conserved interacting residue, Asp110 in the transmembrane III of D_3_R, could be seen in all three ligands‒D_3_R complexes via electrostatic interaction (Figure 11). Other important residues, such as Phe345 and Tyr365, fit tightly into ligands **1**–**3** via *π-π* hydrophobic bonds. Val111 at helix III was also observed forming a *π*-alkyl interaction with both ligands **1** and **3**. Ligands **1**–**3** also interacted with neighboring residues, including His349, Ile183, Thr369, Val86, and Cys114. Similarly, at D_4_R, all the test ligands showed strong interactions with lower binding energies (−12.42 to −9.67 kcal/mol) than the reference drugs (Table 8).

DA, nemonapride, and clozapine were used as the reference agonist and antagonist and had binding energies of −6.1, −13.08, and −10.14 kcal/mol, respectively. 

One of the most crucial residues in stimulating D_4_R, Asp115 interacted with all three ligands in *π*-anion form at helix III, whereas Ser197 interacted only with **1** and **3** on in helix V (Figure 12). Ligand **3** showed an H-bond interaction with Asp115 and Ser196, which is probably why it had the lowest binding energy (−12.42 kcal/mol) among the three ligands. Similarly, Val116, His414, and Leu187 were common interaction sites for three ligands at D_4_R. Other surrounding residues, including Met112, Thr434, Arg186, Phe410, Cys119, and Val193, were involved in hydrophobic interactions with the ligands.

## 3. Discussion

In the present study, we tested Diels–Alder type adducts **1**–**3** from the root bark of *M. alba* and found that they exhibit a mild-to-moderate *h*MAO inhibition effect. The inhibition effect was slightly higher on the *h*MAO-B isozyme (IC_50_: 18.14 to 90.59 µM) than on *h*MAO-A (IC_50_: 54.79 to 114.31 µM). In particular, ligand **1** demonstrated a moderate inhibition effect on the *h*MAO-B isozyme, with an IC_50_ value of 18.14 ± 1.06 µM. Among test compounds **1**–**3**, **1** and **3** are fused benzofurans, and **2** is a mono-isoprenyl substituted flavone. The structural difference between **1** and **3** is the methyl cyclohexene in **1** and methylbenzene in **3**. The methyl cyclohexene group of **1** was involved in specific alkyl interactions with Leu337, Ile335, and Met350 and this moiety is facing toward FAD at the *h*MAO-A active site (Figure 5). However, the methylbenzene of **3** was not involved in any interactions, which explains why **1** had better binding affinity and inhibition potency on *h*MAO-A than **3**. Likewise, in *h*MAO-B inhibition, H-bond interaction of **1** with His115, Pro476, and Glu483 might explain for better binding affinity and activity compared to **3**. In addition, **1** and **2** bound in a similar pose and this might explain the same binding mode for both the ligands. The test ligand activity (*K*_i_ values) did not show a strong correlation with the docking score (binding energies). This variation might be attributed to the physicochemical properties of ligands, especially logP which was predicted high in the range of 6.7 to 7.3 from web-based software PreADMET (v2.0, YONSEI University, Seoul, Korea) (data not reported here). Overall, the results of the *h*MAO inhibition assay reveal that these Diels–Alder type adducts, especially **1**, might have therapeutic value in managing PD. However, this treatment approach is just symptomatic, restoring dopaminergic function in the striatum [29]. Therefore, the discovery of new natural DA agonists is promising.

Depending on their stimulation or inhibition of adenylyl cyclase and modulation of cAMP levels, DA receptors are categorized into two classes: D_1_-like (D_1_R and D_5_R) and D_2_-like (D_2_R, D_3_R, and D_4_R). These DA receptors have specific anatomical distributions and specifically mediate DA action [30]. Several studies have pointed to DA receptor antagonists as a promising approach to managing heroin addiction. For instance, a D_1_R antagonist (SCH 23390) and D_2_R antagonists (haloperidol and raclopride) attenuate heroin-induced reinstatement [31,32], and a D_3_R antagonist (SB-277011A) blocks the acquisition and expression of the conditioned place preference response to heroin [33]. Similarly, a natural alkaloid l-tetrahydropalmatine is a D_1_R/D_2_R antagonist with an anti-addiction property [34], and govadine (D_1_R/D_2_R antagonist) demonstrated antipsychotic properties in conjunction with pro-cognitive effects in rats [35]. The extent of D_2_R binding affinity and antagonizing ability represent the clinical efficacy of antipsychotic drugs [36]. Previously, a root ethanol extract of *M. alba* mediated skin wound healing by upregulating the mRNA levels of chemokine receptor 4, one of the GPCRs [37]. Other than that, no previous studies have reported on GPCR modulation by an *M. alba* root extract or its metabolites.

To evaluate the functional effects of adducts **1**‒**3** on DA (D_1_, D_2_, D_3_, and D_4_) receptors, we conducted a cell-based GPCR functional assay. As shown in Figure 8c,d, **1**–**3** potently and concentration-dependently inhibited the agonist response of DA at D_1_R and D_2L_R. Even at 25 µM, **1**–**3** inhibited the DA response on D_1_R/D_2L_R by 92.32/97.16, 91.09/33.69, and 66.82/81.11%, respectively. Unlike sigmoidal dose–response curves of **1** and **2** at D_1_R, compound **3** showed an unusual non-sigmoidal curve (Figure 8c). A higher standard deviation in response at 50 µM concentration led to unusual appearance. While self-association into colloidal particles at a higher concentration or multi-target actions [38,39,40] explains the possible reason for the observed non-sigmoidal dose–response curve of the compound **3**. Likewise, in the D_2L_R agonist assay, **2** showed nonspecific interference (NSI) in the assay system. This NSI might be attributed to aggregation/colloid formation or chemical reactivity because these are significant sources of nonspecific bioactivity particularly in high throughput screening (HTS) [41]. NSI by aggregates and colloids is detergent sensitive, so it will be confirmed in the coming report. We compared the binding affinity and interacting residues of test compounds **1**–**3** with those of a reference agonist (DA) and antagonist (SCH 23390) via a molecular docking simulation. As shown in Table 5 and Table 6, **1**–**3** showed a high binding affinity (the binding energies of **1**–**3** were lower than those of the reference drugs at D_1_R and D_2_R, except for risperidone at D_2_R). Test ligands **2** shared common H-bond interaction residues, Asp103 and Ser202 with the reference antagonist (SCH 23390). Furthermore, **1**–**3** displayed additional H-bond interactions with serine residues (Ser107, Ser188, and Ser198) and aspartic acid residues (Asp314 and Asp187). Similarly, at D_2_R, **1**–**3** interacted with the key interacting residues Asp114, Trp100, and Phe389 [42]. All three ligands bound to D_2L_R with high affinity and the binding energy was lower than that of the reference antagonist butaclamol. Interactions with Asp114, Cys118, Phe198, Phe389, Trp386, and Tyr416 were common among the test ligands and butaclamol (Table 6). Additional H-bond interactions between Ser197 and **1**, Tyr408 and **2**, and Trp100 and **3** were also observed. Residue Ser197 is a conserved-essential residue within the binding site for binding the D_2_R antagonist risperidone [43], which was also observed for test ligand **2** binding. Tyr408 is located deep in the binding site, whereas Trp100 is at the periphery of the binding site of D_2_R [44], and they were both involved in the binding of test ligands **2** and **3**. According to Salmas et al. [45], Phe389, Phe390, and Trp386 in TM6 are main residues for D_2_R-antagonists. Meanwhile, Phe189, Phe198, and Val190 are necessary as key residues for antagonist ligands binding. Here, Phe389 and Val190 are interacting with ligand **2** whereas Val190 and Phe189 are bound to ligand **3** as hydrophobic bond. Using those findings, we characterized **1**–**3** as D_1_R/D_2L_R antagonists. In a previous study, *M. alba* leaf extract possessed D_2_R-mediated anti-dopaminergic activity, suggesting a possible clinical application for *M. alba* leaves in psychiatric disorders [46]. Our findings suggest that **1**–**3** could have antipsychotic effects.

The test compounds showed an agonist effect on D_3_R and D_4_R. As shown in Figure 8a,b, **1**–**3** showed a potent agonist effect on D_3_R and D_4_R. D_3_R is prominently distributed within the limbic system and mediates the psychiatric manifestation of DA receptor stimulation. Therefore, DA receptor agonists with high affinity for D_3_R have an antidepressant effect [47]. Similarly, Levant et al. suggested that D_3_R-stimulation (rather than D_2_R-stimulation) might mediate the antiparkinsonian effects of DA receptor agonists with a high preference for D_2_R [48]. Rotigotine is an FDA-approved, full DA agonist (rank order: D_3_R >  D_2L_R  >  D_1_R  =  D_5_R  >  D_4.4_R) developed as a transdermal patch for the treatment of PD [1,49].

A previously conducted survey reported that more than 110 patent applications had been submitted concerning selective D_3_R ligands [50]. Unfortunately, none of them has yet received clinical approval due to failures of the pharmacokinetics or safety profiles [51]. Similarly, D_4_R agonism has been implicated in the management of cognitive deficits associated with schizophrenia [52] and attention-deficit/hyperactivity disorder [53] and also to reduce the adverse effects of opioids [54]. 

The results of the functional assays in this study show that the ligands **1**–**3** have concentration-dependent agonist effects on D_3_R and D_4_R (rank order: D_3_R > D_4_R). Even at 25 µM, **1**–**3** showed potent agonist responses on DA D_3_R/D_4_R of 71.92/63.00, 64.99/58.66, and 94.93/‒%, respectively. The agonist effect of **3** on D_4_R was mild (% stimulation of agonist response of 44.85% at 100 µM). The antagonist effect on these receptor subtypes was negligible. We also used molecular docking simulations to compare the binding affinity and interacting residues between test compounds **1**–**3** and D_3_R (Table 7) with those of a reference agonist DA and antagonists (eticlopride and (+)-butaclamol). Likewise, docking simulations of **1**–**3** and D_4_R was compared with those of reference agonists DA and nemonapride, and an antagonist clozapine (Table 8). As tabulated in Table 7 and Table 8, the binding energies of **1**–**3** on D_3_R/D_4_R were comparable to the reference ligands. Interestingly, our prediction demonstrated that they had lower binding energy at D_4_R than at D_3_R. Interaction with Asp110 on D_3_R and Asp115 on D_4_R was in common with the agonist DA. It was reported earlier that a salt-bridge to the carboxylic acid group of the Asp110 on *h*D_3_R and the Asp115 on *h*D_4_R is critical to high-affinity ligand binding to dopaminergic receptors [55]. In this study, though ligands **1**–**3** did not form a salt-bridge with those receptors, they did form strong electrostatic interactions (Pi-Anion). In addition to their electrostatic interactions with Asp115 on D_4_R, **2** and **3** formed H-bond interactions with carboxylic acid group of Asp115.

At a molecular level, D1-like (D_1_ and D_5_) receptor signaling is mediated chiefly by the heterotrimeric G proteins Gα_s/olf_, which cause sequential activation of adenylate cyclase, cyclic AMP-dependent protein kinase, and the protein phosphatase-1 inhibitor DARPP-32 [56]. A recent study showed that hypersensitivity of D_1_R is responsible for l-DOPA-induced activation of mTORC1 signaling, and D_1_R antagonist (SCH23390) blocked the l-DOPA-induced phosphorylation of p70 S6 kinase (S6K), ribosomal protein S6, and eukaryotic translation initiation factor 4E (eIF4E)-binding protein 1 (4E-BP1) in 6-OHDA–lesioned mice [57]. Moreover, DA through D_1_R induces ERK stimulation via a cAMP/protein kinase A (PKA)/*Rap1*/*B-Raf*/MAPK/ERK kinase (MEK) pathway and SCH 23,390 completely blocks the p-ERK1/2 levels induced by DA [58].

Likewise, D2-like (D_2_, D_3_, and D_4_) receptor signaling is mediated by the heterotrimeric G proteins Gα_i/o_, which causes inhibition of adenylate cyclase thereby decreasing the phosphorylation of PKA substrates. Binding of DA to DA receptors regulate signaling via cAMP response element-binding protein (CREB), glutamate receptors, GABA receptors, and ion channels (e.g., calcium and potassium) [59]. Previous study reports that stimulation of D2-like receptors decreases PKA-stimulated phosphorylation of DARPP-32 at Thr34 and increases phosphorylation at Thr75 [60,61]. Even though DARPP-32 is an important modulator and/or effector of DA receptors signaling, it is not the only modulator of DA-mediated activities [62]. The test compounds of the present study showed a unique profile, i.e., moderate *h*MAO inhibition with good D_1_R/D_2L_R antagonist and D_3_R/D_4_R agonist effect. So, what could be the underlying mechanism and in vivo effect is very interesting and need to be studied shortly.

## 4. Materials and Methods

### 4.1. Chemicals and Reagents 

Mulberrofuran G (**1**), kuwanon G (**2**), and albanol B (**3**) were isolated and identified from the root bark of *M.* alba Linn following a method described previously [63]. The purity of these compounds was considered to be >98% as evidenced by spectral data. A MAO-GloTM assay kit was purchased from Promega (Promega Corporation, Madison, WI, USA). Transfected Chinese hamster ovary (CHO) cells were obtained from Eurofins Scientific (Le Bois I’Eveque, France). Hank’s balanced salt solution (HBSS), Dulbecco’s modified Eagle medium, and 4-(2-hydroxyethyl)-1-piperazineethanesulfonic acid (HEPES) buffer were obtained from Invitrogen (Carlsbad, CA, USA). The *h*MAO isozymes and reference drugs selegiline, DA, serotonin, butaclamol, SCH 23390, clozapine, and (S)-WAY-100635 were purchased from Sigma-Aldrich (St. Louis, MO, USA).

### 4.2. In Vitro Human MAO Inhibition and Enzyme Kinetics

The potential of the test compounds for human MAO inhibition was evaluated via a chemiluminescence technique using the MAO-Glo kit (Promega, Madison, WI, USA). Detailed experimental conditions and procedures were reported previously [64,65]. The test compounds were evaluated at a concentration of 6, 30, and 120 µM. Selegiline was used as a positive control. The kinetic analysis of *h*MAO inhibition was analyzed at different concentrations of *h*MAO substrate depending on the isozyme (40, 80, and 160 µM for *h*MAO-A and 4, 8, and 16 µM for *h*MAO-B) following the same method of enzyme inhibition. The concentrations of the test compounds for the kinetic study are presented in Figure 2 and Figure 3. Kinetic parameters were analyzed using SigmaPlot (v12.0, SPP Inc., Chicago, IL, USA).

### 4.3. Cell-Based Functional GPCR Assay

Cell-based functional GPCR assays were conducted in CHO cells transfected with a plasmid containing the GPCR gene of interest. The functional activity of the test compounds (agonist or antagonist) was evaluated by measuring their effects on cAMP modulation or Ca^2+^ ion mobilization, depending on the receptor type. All assays were performed at Eurofins Cerep (Le Bois I’Eveque, France) following their in-house protocol, as stated in our previous reports [66,67,68].

### 4.4. Measurement of cAMP Level

The functional activity of the test compounds on D_1_R, D_3_R, and D_4_R was assessed by evaluating the effect on cAMP modulation. For this, stable transfectants (CHO-D_1_R, CHO-D_3_R, and CHO-D_4_R) were suspended in HBSS (Invitrogen, Carlsbad, CA, USA) containing 20 mM HEPES buffer and 500 μM 3-isobutyl-1-methylxanthine, distributed into microplates (5 × 103 cells/well), and incubated for 30 min at room temperature (RT) in the absence (control) or presence of the test compounds (6.25, 12.5, 25, 50, and 100 μM) or reference agonist (DA). In the D_3_R and D_4_R assays, the adenylyl cyclase activator NKH 477 was added at a final concentration of 1.5 and 0.7 μM and incubated for 30 and 10 min, respectively, at 37 °C. Then, the cells were lysed and a fluorescence acceptor (D2-labeled cAMP) and fluorescence donor (anti-cAMP antibody with europium cryptate) were added. The fluorescence transfer was measured at λex = 337 nm and λem = 620 and 665 nm using a microplate reader (Envision, Perkin Elmer, Waltham, MA, USA) after 60 min of incubation at RT. Agonist effects are expressed as the % of the control response to 10 μM DA for D_1_R and 300 nM DA for D_3_R/D_4_R. Similarly, antagonist effects are expressed as the % inhibition of the control response to DA 300 nM for D1R, 10 nM for D_3_R, and 100 nM for D_4_R. The reference agonist DA and antagonists SCH 23390, (+)-butaclamol, and clozapine were used to validate the study.

### 4.5. Measurement of Intracellular [Ca^2+^] Levels

The functional activity of the test compounds on D_2_R was tested by fluorimetrically evaluating their effect on cytosolic Ca^2+^ ion mobilization. In brief, CHO-D_2L_R cells were separately suspended in HBSS (Invitrogen, Carlsbad, CA, USA) complemented with 20 mM HEPES buffer and distributed into microplates (1 × 105 cells/well). Then, a fluorescent probe (Fluo8, AAT Bioquest) mixed with probenecid in HBSS (Invitrogen, Carlsbad, CA, USA) supplemented with 20 M HEPES (Invitrogen) (pH 7.4) was added to each well, and the cells were allowed to equilibrate for 60 min at 37 °C. Thereafter, the plates were positioned in a microplate reader (FlipR Tetra, Molecular Device), and compounds **1**–**3** (6.25, 12.5, 25, 50, and 100 μM), reference agonist, or HBSS (basal control) were added. We then measured the fluorescent intensity, which varied in proportion to the free cytosolic Ca^2+^ ion concentration. Agonist effects are expressed as the % of the control response to 10 μM DA. Similarly, antagonist effects are expressed as the % inhibition of the control response to 700 nM DA. Reference agonist (DA) and antagonist (butaclamol) were used to validate the study.

### 4.6. Homology Modeling

The primary sequence of the human DA D_1_ receptor was obtained from UniProt (ID: P21728, DRD1_HUMAN). The β_2_R (β2 adrenergic receptor) has a higher similarity to DA D_1_R in the binding site region and sequence identity [28]. Hence, the model was built on the template of the β_2_R crystal structure from the RCSB protein data bank (PDB) using ID 2RH1 with SWISS-MODEL. Refining the model was conducted using the ModRefiner sever [69].

### 4.7. In Silico Molecular Docking Simulation

Automated single docking simulations were carried out with AutoDock 4.2 [70]. X-ray crystallographic structures of *h*MAO-A, *h*MAO-B, *h*D_2L_R, *h*D_3_R, and *h*D_4_R were obtained from the PDB with IDs 2BXR, 2BYB, 6CM4, 3PBL, and 5WIV, respectively. The 3D chemical structures of the three test compounds were obtained from PubChem Compound (NCBI, CIDs 196583, 5281667, and 480,819 for compounds **1**–**3**, respectively). The crystal structures of the reference compounds, selegiline, harmine, DA, SCH 23390, risperidone, butaclamol, eticlopride, nemonapride, and clozapine were also obtained from NCBI under CIDs 26758, 5280953, 681, 5018, 5073, 37461, 57267, 156333, and 135398737, respectively. Water and ligand molecules were removed using Discovery Studio (v17.2, Accelrys, San Diego, CA, USA). In the case of the *h*MAO isozymes, the cofactor flavin adenine dinucleotide (FAD) was retained. The Lamarckian genetic algorithm method in AutoDock 4.2 was applied. For the docking calculations, Gasteiger charges were added by default, and all the torsions were allowed to rotate. The grid maps were generated with the AutoGrid program. The docking protocol for rigid and flexible ligand docking consisted of 10 independent genetic algorithms, and other parameters were set using the defaults in the AutoDock Tools. The docking results were visualized using Discovery Studio.

## 5. Conclusions

This study is the first to report the therapeutic potential of natural Diels–Alder type adducts, mulberrofuran G (**1**), kuwanon G (**2**), and albanol B (**3**) from *M. alba* root bark in neurodegenerative diseases. Our investigations identified **1**–**3** as novel multi-target-directed ligands for the management of neurodegenerative diseases via *h*MAO inhibition and dopaminergic receptor modulation. Specifically, cell-based GPCR functional assays in vector-transfected CHO cells expressing DA receptors characterized **1**‒**3** as potent D_1_R/D_2L_R antagonists and D_3_R/D_4_R agonists. The assay results were further supported by molecular docking studies, which predicted tight binding between the test ligands and the receptors. Overall, the results of this study provide evidence that ligands **1**‒**3** from *M. alba* could be developed into neuronal drugs targeting DA receptors. Further in vivo studies are warranted to fully and precisely characterize the mechanism of action via a signal transduction pathway.

## Figures and Tables

**Table 1:** Human monoamine oxidase (*h*MAO) inhibitory potential of compounds from *Morus alba.*

Compounds	Human Monoamine Oxidase A (*h*MAO-A)		
	IC_50_ (μM, Mean ± SD) ^a^	*K_i_* Value ^b^	Inhibition Type ^c^
**1**	54.79 ± 0.03	26.96 ± 3.98	Competitive
**2**	70.16 ± 2.60	28.29 ± 2.02	Competitive
**3**	114.31 ± 2.30	46.93 ± 4.12	Competitive
Selegiline ^d^	12.51 ± 1.11	*NT*	*NT*
Harmine ^d, e^	0.006 [26]	*NT*	*NT*
	**Human Monoamine Oxidase B (*h*MAO-B)**		
**1**	18.14 ± 1.06	17.01 ± 3.31	Noncompetitive
**2**	57.71 ± 2.12	52.09 ± 5.56	Noncompetitive
**3**	90.59 ± 1.72	55.19 ± 7.79 ^f^/186.2 ± 10.26 ^g^	Mixed
Selegiline ^d^	0.30 ± 0.01	*NT*	*NT*
Safinamide ^d,e^	0.00512 [27]	*NT*	*NT*

NT: Not tested. ^a^ The 50% inhibitory concentration (IC_50_) values (μM) were calculated from a log dose-inhibition curve and expressed as the mean ± SD of triplicate experiments. ^b^ The *h*MAO inhibition constant (*K*_i_) was determined using a Dixon plot. ^c^ The *h*MAO inhibition type was determined using Lineweaver–Burk plots and Dixon plots. ^d^ Reference inhibitor. ^e^ Values extracted from the literature. ^f, g^ *K*_ic_ and *K*_iu_ values, respectively.

**Table 2:** Binding site residues and docking scores of **1**–**3** and reference inhibitors in human monoamine oxidase A (*h*MAO-A) (2BXR) obtained using Autodock 4.2.

Compound	Binding Energy (kcal/mol) ^a^	H-bond Interacting Residues ^b^	Hydrophobic Interacting Residues ^b^	Electrostatic Interacting Residues ^b^
**1**	−9.54	Gly110, Thr336, Ile207, Gly214, Ser209	Val210 (Pi-Sigma, Pi-Alkyl), Ile325 (Pi-Sigma), Phe208 (Pi-Pi Stacked, Pi-Pi T-Shaped), Ile358 (Alkyl), Leu337 (Alkyl), Ile335 (Alkyl), Met350 (Alkyl), Val93 (Pi-Alkyl),	-
**2**	−6.74	Met300, Leu298, Asp359, Gly404, Cys398, Trp397, Glu400	Ala302 (Pi-Alkyl, Alkyl)	-
**3**	−8.62	Gln296, Ile295, Gly404, Tyr410, Met300, Thr183, Ser184	Pro299, Ala279, Ala302 (Pi-Alkyl)	Glu188 (Pi-Anion)
Selegiline	−6.54	-	Ile335 (Pi-Sigma), Leu337 (Pi-Alkyl), FAD600 (Pi-Alkyl), Tyr407 (Pi-Alkyl), Tyr444 (Pi-Alkyl)	-
HRM ^c^(Harmine)	−6.46	FAD600	Tyr444 (Pi-Sigma), FAD600 (Pi-Sigma, Pi-Pi T-shaped, Pi-Alkyl), Tyr444 (Pi-Pi Stacked), Phe352 (Pi-Pi T-shaped), Tyr407 (Pi-Alkyl), Ile335 (Pi-Alkyl)	-

^a^ Estimated binding free energy of the ligand–receptor complex. ^b^ The number of hydrogen bonds and all amino acid residues from the enzyme–inhibitor complex was determined with the AutoDock 4.2 program. ^c^ 7-Methoxy-1-methyl-9H-pyrido [3,4-b]indole.

**Table 3:** Binding site residues and docking scores of **1**–**3** and reference inhibitors in human monoamine oxidase B (*h*MAO-B) (2BYB) obtained using Autodock 4.2.

Compound	Binding Energy (kcal/mol) ^a^	H-bond Interacting Residues ^b^	Hydrophobic Interacting Residues ^b^	Electrostatic Interacting Residues ^b^
**1**	−11.09	His115, Pro476, Glu483	Phe103 (Pi-Pi Stacked, Pi-Pi T-shaped, Pi-Alkyl), Val106 (Pi-Alkyl), Ile477 (Pi-Alkyl)	Glu483(Pi-Anion)
**2**	−12.65	Pro104, Asn116, Glu483, Phe103, Thr478	Tyr112 (Pi-Sigma), Phe103 (Pi-Pi Stacked), Val106 (Alkyl, Pi-Alkyl), Pro102 (Alkyl, Pi-Alkyl), Tyr112 (Pi-Alkyl), Trp119 (Pi-Alkyl), Pro104 (Pi-Alkyl), Leu164 (Pi-Alkyl)	Glu483(Pi-Anion)
**3**	−10.05	Thr195, Pro104, Asn116, Thr478, Gly193	Ile477 (Pi-Sigma), Trp119 (Pi-Pi Stacked), Phe103 (Pi-Pi T-shaped), Thr195 (Amide-Pi Stacked), Gly194 (Amide-Pi Stacked), Arg120 (Alkyl, Pi-Alkyl), Val106 (Pi-Alkyl)	Asp123(Pi-Anion), Glu483(Pi-Anion)
Selegiline ^c^	−7.06	Ile198	Tyr398 (Pi-Pi Stacked), Tyr435 (Pi-Pi Stacked), FAD600 (Pi-Pi T-shaped), Leu171 (Alkyl), Cys172 (Alkyl), Phe188 (Pi-Alkyl)	-
Safinamide ^c^	−9.86	Cys172, Ile199, Tyr326, Thr201	Leu171 (Pi-Sigma, Pi-Alkyl), Tyr398 (Pi-Pi Stacked), Tyr326 (Pi-Pi T-shaped), Ile199 (Pi-Alkyl)	-

^a^ Estimated binding free energy of the ligand–receptor complex. ^b^ The number of hydrogen bonds and all amino acid residues from the enzyme–inhibitor complex were determined with the AutoDock 4.2 program. ^c^ Reference inhibitors.

**Table 4:** Efficacy values (% stimulation and % inhibition) of Diels–Alder type adducts (**1**–**3**) from *M. alba* on DA (D_1_, D_2L_, D_3_, and D_4_) receptors.

Receptors	1	2	3	Reference Drugs
	% Stimulation ^a^ (% Inhibition ^b^)	% Stimulation ^a^ (% Inhibition ^b^)	% Stimulation ^a^ (% Inhibition ^b^)	EC_50_^c^ (IC_50_^d^)
D_1_ (h)	17.2 ± 8.4 (87.65 ± 1.19)	0.85 ± 0.24 (98.85 ± 1.79)	*INTER* (67.80 ± 9.05)	28 (2.8)
D_2L_ (h)	7.10 ± 1.47 (101.30 ± 0.16)	*NSI* (99.15 ± 0.77)	4.10 ± 1.06 (78.55 ± 3.61)	12 (28)
D_3_ (h)	119.9 ± 2.44 (−28.7 ± 11.15)	124.3 ± 0.76 (−27.4 ± 7.79)	102.8 ± 1.36 (−13.4 ± 1.87)	4.1 (20)
D_4_ (h)	86.30 ± 0.99 (−20.8 ± 6.93)	90.45 ± 0.14 (−29.6 ± 7.21)	46.10 ± 1.76 (26.9 ± 5.09)	21 (150)

^a, b^ % Stimulation and % inhibition, respectively, of control agonist response at 100 µM of test compounds. ^c^ EC_50_ (nM) values of standard agonist DA. ^d^ IC_50_ (nM) values of standard antagonists (D_1_: SCH-23390, D_2L_: butaclamol, D_3_: (+)-butaclamol, D_4_: clozapine. *INTER*: Test compound interfered with the assay detection method. *NSI*: Test compound interfered nonspecifically in the assay.

**Table 5:** Binding sites and docking scores of compounds on *h*D_1_R.

Target	Compounds	Binding Energy (kcal/mol)	H-bond Interaction Residues	Hydrophobic Interacting Residues	Electrostatic Interacting Residues
*h*D_1_R	Dopamine ^a^ (agonist)	−5.59	Asp103 (Salt bridge), Ser202, Asn292, Ser199	Phe289 (Pi-Pi T-shaped), Ile104 (Pi-Alkyl)	Phe288(Pi-Cation)
	SCH23390 ^a^ (antagonist)	− 6.94	Asp103 (Salt bridge), Ser199, Ser202	Leu190 (Pi-sigma), Phe288 (Pi-Pi T-shaped), Ile104 (Pi-Alkyl), Ala195 (Pi-Alkyl)	-
	**1**	−9.22	Lys81, Leu291, Asp314, Ser188	Leu295 (Pi-sigma), Phe313 (Pi-Pi Stacked), Phe306 (Pi-Pi T-shaped), Ser188 (Amide-Pi Stacked), Leu295 (Pi-Alkyl), Leu291 (Pi-Alkyl)	Lys81(Pi-Cation), Asp314(Pi-Anion)
	**2**	−7.1	Lys81, Ser107, Ser202, Asp187, Asp103, Ser198	Val100 (Pi-sigma), Val317 (Pi-Sigma, Pi-Alkyl), Phe313 (Pi-Pi T-shaped), Leu190 (Alkyl), Cys186 (Alkyl), Phe288 (Pi-Alkyl), Ile104 (Pi-Alkyl)	Asp187 (Pi-Anion)
	**3**	−9.2	Asp187, Ser188	Asp187 (Pi-Sigma). Leu295 (Pi-Sigma, Pi-Alkyl), Phe30 6 (Pi-Pi T-shaped), Pro171 (Pi-Alkyl), Arg192 (Pi-Alkyl), Ala195 (Pi-Alkyl)	-

^a^ Reference ligand for hD_1_R.

**Table 6:** Binding sites and docking scores of compounds on *h*D_2L_R.

Target	Compounds	Binding Energy (kcal/mol)	H-bond Interaction Residues	Hydrophobic Interacting Residues	Electrostatic Interacting Residues
*h*D_2L_R	Dopamine ^a^ (agonist)	−6.98	Asp114 (Salt bridge), Tyr416, Thr119	Trp386 (Pi-Pi T-shaped), Val115 (Pi-Alkyl)	-
	Risperidone ^a^ (agonist)	−12.7	Asp114 (salt bridge), Thr119	Trp100 (Pi-Pi T-shaped, Pi-Alkyl), Trp386 (Pi-Pi T-shaped), Val91(Alkyl), Leu94 (Alkyl), Val115 (Alkyl, Pi-Alkyl), Val111 (Alkyl), Ile184 (Alkyl), Phe110 (Pi-Alkyl), Phe389 (Pi-Alkyl), Cys118 (Pi-Alkyl), Ala122 (Pi-Alkyl)	-
	Butaclamol ^a^ (antagonist)	−6.9	Asp114 (Salt bridge), Ser193	Phe389 (Pi-Pi Stacked, Pi-Pi T-shaped, Pi-Alkyl), Tyr416 (Pi-Pi Stacked), Cys118 (Alkyl), Phe198 (Pi-Alkyl), Trp386 (Pi-Alkyl), Phe390 (Pi-Alkyl)	-
	**1**	−8.11	Ser197, Asp114, Thr412,	Thr412 (Pi-Sigma), Phe110 (Pi-Sigma), Trp110 (Pi-Pi T-shaped, Pi-Alkyl), Trp386 (Pi-Pi T-shaped), Tyr416 (Pi-Pi- T-shaped), Val111 (Alkyl), Ile184 (Alkyl),	Asp114 (Pi-Anion)
	**2**	−8.23	Asn396, Tyr408, Ile184	Tyr408 (Pi-Pi Stacked), Tyr100 (Pi-Pi T-shaped), Phe389 (Pi-Alkyl), Tyr416 (Pi-Alkyl), Ile184 (Pi-Alkyl), Val190 (Pi-Alkyl)	-
	**3**	−10.45	Trp100, Cys118, Ser193, Asp114	Ile184 (Pi-Sigma, Alkyl), Trp100 (Pi-Pi T-shaped), Trp386 (Pi-Pi T-shaped), Val190 (Alkyl), Phe189 (Pi-Alkyl), Val115 (Pi-Alkyl)	Asp114 (Pi-Anion)

^a^ Reference ligand for *h*D_2L_R.

**Table 7:** Binding sites and docking scores of compounds on *h*D_3_R.

Target	Compounds	Binding Energy (kcal/mol)	H-bond Interaction Residues	Hydrophobic Interacting Residues	Electrostatic Interacting Residues
*h*D_3_R	Dopamine ^a^ (agonist)	−5.72	Asp110 (Salt bridge), Tyr373, Val111, Thr115, Ser196	Val111 (Pi-Alkyl), Cys114 (Pi-Alkyl)	
	Eticlopride ^a^ (antagonist)	−9.22	Asp110 (Salt bridge), Tyr373	Phe345 (Pi-Pi T-shaped), Ile183 (Alkyl, Pi-Alkyl), Val189 (Alkyl), VAl111 (Pi-Alkyl)	
	(+)-butaclamol ^a^ (antagonist)	−10.69	Asp110(Salt bridge)	Val111 (Alkyl), Cys114 (Alkyl), Trp342 (Pi-Alkyl), Phe345 (Pi-Alkyl), Phe346 (Pi-Alkyl), Val86 (Pi-Alkyl)	
	**1**	−5.89	Tyr365, Cys181, Ser366, Thr369	Ile183 (Pi-Sigma), Phe345 (Pi-Pi T-shaped), His349 (Pi-Pi T-shaped), Tyr365 (Pi-Pi T-shaped), Val86 (Alkyl, Pi-Alkyl), Leu89 (Alkyl), Phe106 (PI-Alkyl), Val107 (Pi-Alkyl), Val111 (Pi-Alkyl)	Asp110 (Pi-Anion)
	**2**	−7.45	Tyr365, Thr369, Cys181,	Thr369 (Pi-Sigma), Phe345(Pi-Pi Stacked, Pi-Alkyl), Phe106 (Pi-Pi T-shaped), Tyr365 (Pi-Pi T-shaped), Val86 (Alkyl), Leu89 (Alkyl), Phe346 (Pi-Alkyl), Val107 (PI-Alkyl)	Asp110 (Pi-Anion),
	**3**	−10.41	Ile183, Val110, Thr115	Leu89 (Pi-Sigma), Thr359 (Pi-Sigma), Phe345 (Pi-Pi Stacked), Tyr365 (PI-Pi T-shaped), Val86 (Alkyl, Pi-Alkyl), Tyr36 (Pi-Alkyl), Val111 (Pi-Alkyl), Cys114 (Pi-Alkyl),	Asp110 (Pi-Anion)

^a^ Reference ligand for *h*D_3_R.

**Table 8:** Binding sites and docking scores of compounds on *h*D_4_R.

Target	Compounds	Binding Energy (kcal/mol)	H-bond Interaction Residues	Hydrophobic Interacting Residues	Electrostatic Interacting Residues
*h*D_4_R	Dopamine ^a^ (agonist)	−6.1	Asp115(Salt bridge), Thr120, Ser196, Tyr438	Cys119(Pi-Alkyl), Val116(Pi-Alkyl), Phe411(Pi-Pi T-shaped)	
	Nemonapride ^a^ (agonist)	−13.08	Asp115(Salt bridge), Tyr438, Ser196	Val116 (Pi-Sigma), Phe91 (Pi-Pi T-shaped), Phe410 (Pi-Pi T-shaped), Leu90 (Amide-Pi Stacked), Val193 (Alkyl), Leu111 (Pi-Alkyl)	
	Clozapine ^a^ (antagonist)	−10.14	Asp115(Salt bridge)	Leu187(Pi-Sigma), Phe410(Pi-PI T-shaped), His414(Pi-Pi T-shaped), Val116(Alkyl, Pi-Alkyl), Val193(Pi-Alkyl)	
	**1**	−9.67	Ser196, Leu187, Val430, Thr434	Val116 (Pi-Sigma, Pi-Alkyl), Leu187 (Pi-Sigma), Thr434 (Pi-Sigma), Phe411 (Pi-Pi T-shaped), His414 (PI-Pi T-shaped), Phe410 (Pi-Pi T-shaped), Met112 (Alkyl), Cys185 (Alkyl), Cys119 (Alkyl, Pi-Alkyl), Arg186 (Pi-Alkyl)	Asp115 (Pi-Anion)
	**2**	−10.34	Ser197, Thr434, Asp115, Tyr438	Val193 (Pi-sigma), His414 (Pi-Pi Stacked, Pi-Pi T-shaped), Met112 (Alkyl), Leu187 (Alkyl, Pi-Alkyl), Phe91 (Pi-Alkyl), Arg186 (Pi-Alkyl),Val116 (Pi-Alkyl)	Asp115 (Pi-Anion)
	**3**	−12.42	Leu187, Asp115, Ser196	Leu187 (Pi-Sigma, Alkyl, Pi-Alkyl), Phe410 (Pi-Pi T-shaped), His414 (Pi-Pi T-shaped, Pi-Alkyl), Val193 (Alkyl, Pi-Alkyl), Val116 (Pi-Alkyl)	Asp115 (Pi-Anion)

^a^ Reference ligand for *h*D_4_R.
